# PeriSense: Ring-Based Multi-Finger Gesture Interaction Utilizing Capacitive Proximity Sensing

**DOI:** 10.3390/s20143990

**Published:** 2020-07-17

**Authors:** Mathias Wilhelm, Daniel Krakowczyk, Sahin Albayrak

**Affiliations:** DAI-Labor, Technische Universität Berlin, 10587 Berlin, Germany; mathias.wilhelm@dai-labor.de (M.W.); daniel.krakowczyk@dai-labor.de (D.K.)

**Keywords:** capacitive sensing, ring, finger augmentation device, wearable interaction device, finger gesture recognition

## Abstract

Rings are widely accepted wearables for gesture interaction. However, most rings can sense only the motion of one finger or the whole hand. We present PeriSense, a ring-shaped interaction device enabling multi-finger gesture interaction. Gestures of the finger wearing ring and its adjacent fingers are sensed by measuring capacitive proximity between electrodes and human skin. Our main contribution is the determination of PeriSense’s interaction space involving the evaluation of capabilities and limitations. We introduce a prototype named PeriSense, analyze the sensor resolution at different distances, and evaluate finger gestures and unistroke gestures based on gesture sets allowing the determination of the strengths and limitations. We show that PeriSense is able to sense the change of conductive objects reliably up to 2.5 cm. Furthermore, we show that this capability enables different interaction techniques such as multi-finger gesture recognition or two-handed unistroke input.

## 1. Introduction

Finger rings are a widely used and accepted article of jewelry in our culture, carrying symbolic and mythical meaning. Due to their small size and form factor, they can be worn all day without being obtrusive or drawing much attention. Equipping a ring with sensors can realize a ubiquitous and unobtrusive gesture interaction device. Therefore, it is no surprise that sensor rings gain more and more interest in the human–computer interaction community [1]. This increased interest already led to first elementary consumer products, e.g., Motiv Ring [2] or Jakcom R3F Smart Ring [3] which sense different activities and control commands as well as providing haptic and visual feedback.

As stated in [1], current approaches to ring-based gesture interaction lack a broad interaction space, which limits the variety of applications. This is because those rings are only able to sense motion or bend angle of a single finger. Possible solutions to this problem are the use of multiple rings [4] or rings spanning multiple fingers [5]. Both reduce the obtrusiveness and comfort, and thus the suitability for daily use.

**Figure 1:**
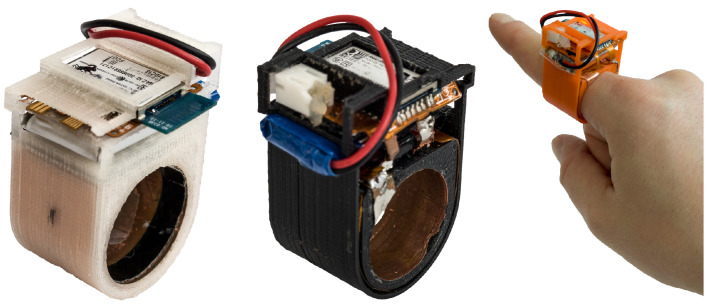
PeriSense prototypes in different colors and sizes.

In this work, we present PeriSense (Figure 1), a single ring enabling a broad range of multi-finger interactions. It utilizes capacitive sensing to measure the approximate distances to other fingers. The paper focuses on the evaluation of the capabilities and limitations of PeriSense in the application of finger gesture recognition. We expect that using capacitive sensing enables a broad interaction space involving multiple fingers as known from other devices like camera-based approaches or data gloves.

The key contributions of this work are listed below.

- A concept of using capacitive sensing to enable multiple finger gesture interaction with a single ring, showcased by a prototype, called PeriSense.
- Evaluations demonstrating the interaction space of the prototype:
  – A study determining the range of resolution at different distances.
  – An evaluation studying the strengths and weaknesses based on two different interaction techniques.

The following section describes the state-of-the-art of wearables in hand and finger gesture recognition. Afterwards, Section 3 describes the working principles and system architecture of PeriSense. This section also evaluates the sensor resolution. Section 4 studies finger gesture interaction and two-handed unistroke input regarding the interaction space. Section 5 discusses some potential use cases and applications for PeriSense. Finally, Section 6 concludes this paper by disclosing limitations and proposing future work.

## 2. Related Work

Wearable interaction devices undergo a continuously increasing popularity in the HCI community and even in the consumer market [1,6]. This led to a vast variety of different wearables. As PeriSense is a wearable in the form of a ring and as its concept addresses finger interaction, we focus on analyzing the state-of-the-art regarding finger-worn wearables, and afterward wearables enabling finger interaction in general. Finally, we discuss our preliminary work on this topic and describe the distinctions to these.

### 2.1. Finger-Worn Wearables Enabling Finger Interaction

Rings are probably the largest category of interaction devices worn on the finger. Most rings are equipped with motion sensors, such as an accelerometer (to sense acceleration), gyroscope (to sense the change of orientation), or magnetometer (to sense the relative orientation with respect to the earth), in order to sense gestures and finger taps (cf. [4,7,8,9,10,11,12,13,14]).

Another common type of sensors are magnetometers sensing the influence of permanent magnets in the close-proximity environment, such as uTrack [15]. There, two magnetometers are worn on the ring finger, enabling 3D input by wearing a magnet on the thumb. A similar configuration is applied by SynchroWatch [16], where a magnet is worn on a thumb ring and a magnetometer of a smartwatch is used to sense the extension and reposition of the thumb in order to control smartwatch applications. In contrast to these approaches, Simmons and Welsh [17] placed a magnetometer on each finger and a magnet on the back of the hand. This configuration allows accurate finger tracking, but it requires equipping the whole hand. Parizi et al. [18] follows a similar approach with AuraRing, where instead of a magnet a coil is embedded in the ring. Several coil sensors around the wrist measure the magnetic field, from which the finger position is determined.

FingerSound [19] and FingOrbit [20] enable unistroke gestures drawn with the thumb on the palm. They are worn on the thumb and are equipped with a piezo-microphone recording the sound when the thumb is moving over the palm. Additionally, a gyroscope is used to track the directional changes of the thumb. This configuration allows a high accuracy recognition of Graffiti-style letters.

Many ring devices consist of buttons or touch areas for binary input. OctaRing [21], for example, consists of eight touch areas for pressure-sensitive multi-touch input enabling complex input patterns. Thumb-In-Motion [22] utilizes a capacitive touch matrix on a ring in order to enable thumb slides and thumb tap recognition on the ring. Nenya [23], in contrast, recognizes twists and slides of the ring along the finger.

To the best of our knowledge, there exist only three rings detecting the movement of multiple fingers. TypingRing [13] detects finger taps on a surface in order to enable text input. It uses proximity sensors in order to detect if the neighboring finger is next to the ring or not.

The prototype eRing [24] utilizes capacitive sensing too, but can only detect if an electrode is in very close proximity (< 10 mm) to a neighboring finger or not. Furthermore, the applied sensing approach is very slow. The measurement of the loading and unloading time of the electrodes is in the worst case about 1 Hz and on average less 10 Hz. Based on this input technique the authors defined two multi-finger gestures sets where each gesture produced a unique pattern. However, the gesture sets were only tested with a couple of examples from one author of the paper. Furthermore, their ring prototype is not self-contained and connects with a computer via a USB cable.

CyclopsRing [25] utilizes a fish-eye camera in order to enable manifold finger interactions. However, the camera performs poorly under fast and intense changes in illumination. Furthermore, it is much more challenging to implement a resource-friendly image transmission and the corresponding gesture recognition approach on a mobile phone, not to mention on the ring itself.

Furthermore, there exist a couple of wearable interaction devices sensing (multiple) finger gestures beside finger rings. Most notable are nail covers. They have similar capabilities such as ubiquity and unobtrusiveness. While Nail+ [26] can only detect different force touch interactions, NailIO [27] can distinguish on-nail finger swipe gestures. Another approach is textile finger worn input devices such as TIMMi [28]. It is worn on the index finger to measure its finger bend and recognizes touch events with the thumb on TIMMi itself using a conductive elastomer.

Rings offer many possibilities for the recognition of finger gestures. However, most rings can only detect the movement and gestures of a finger or the whole hand. They cannot detect, for example, whether all fingers are formed into a fist or only one or two fingers are bent. Only the CyclopsRing can detect complex multi-finger gestures. However, it uses a camera. This prevents objects from being held in hand during the interaction, the power supply is limited to less than a few hours, and it can cause privacy concerns for the user.

### 2.2. Arm and Body Worn Wearables Enabling Finger Interaction

Another obvious wearable enabling multiple finger interaction is a data glove (cf. [29,30,31,32]). It allows the equipment of a multitude of sensors, enabling it to detect and track the finger motions and touch interactions very precisely. However, a glove lacks in comfort and practicability for everyday use and is thus more suitable for particular purposes such as text input for head-mounted displays or accurate machine control. A data glove that also uses capacitive sensing is HandSense [33].

Wristbands are also very popular and well-accepted wearables. Most technologies can also be integrated into a smartwatch. Some of them are even able to detect finger movements and tips. A powerful and precise approach is to mount a camera, such as that presented by Ahmad and Msilek [34], Kim et al. [35], Sridhar et al. [36], Yeo et al. [37], and Wang et al. [38,39]. However, cameras mounted on the wrist have to deal with occlusion caused by long clothes. Furthermore, they require an increased demand for resources for image transmission and processing. BeamBand [40] uses an array of ultrasonic sensors mounted on a wrist band to detect different hand gestures. However, it is confronted with similar problems as the camera-based approaches. Dementyev and Paradiso [41], Fukui et al. [42], McIntosh et al. [43], and Rekimoto [44] use a wristband to detect finger movements by measuring changes in the arm contour caused by the displacement of the bones and chords during the movement. Zhang and Harrison [45] measure these variations in the arrangement of chords and bones using tomography, McIntosh et al. [46] and Ortega-Avila et al. [47] by infrared lights, and McIntosh et al. [48] by ultrasound imaging. Moreover, electromyography measuring the electrical activity produced by muscles in the forearm can be used to detect and distinguish finger movements as shown by Amma et al. [49], Haque et al. [50], and Huang et al. [51]. Kato [52] and Yokota [53] used a high-frequency sound that is induced into the forearm. Hand and finger movements cause a frequency shift of the induced sound which can be applied to recognize finger gestures. In the opposite case, SoundCraft [54] uses microphones integrated into a smartwatch to record environmental sounds, e.g., moving the finger over a desk, which is then utilized for hand gesture and interaction recognition. Laput and Harrison [55] detect fine-grained hand activities using high-speed acceleration data from a Smartwatch.

Despite having advantages such as general acceptance, unobtrusiveness, and the possibility of integration into smartwatches, these devices cannot sense subtle finger gestures, are partially uncomfortable in the case of pressure-based arm contour measurement devices, or require too many resources in case of camera-based devices.

### 2.3. Distinction from Our Preliminary Work

In our first preliminary work, with a very simple prototype we tested whether capacitive sensing is basically suitable for the recognition of multi-finger gestures with a ring. In this work, we present a new prototype and its evaluation, which differs significantly from eRing in the following points.

- **Sensing Method:** eRing measures the time required to charge the electrodes with a direct current. If a finger is close to the electrode, charging will take longer. In the worst case, it is up to 1s. The average rate is less than 10 Hz. PeriSense, on the other hand, uses a high-frequency voltage and measures the change in the alternating field to determine the capacitance. This allows a transmission rate of 100 Hz to be achieved. Additionally, PeriSense has an increased effective sensing range. Moreover, PeriSense is also equipped with a motion sensor.
- **Noise Handling:** No noise reduction methods were implemented in eRing. PeriSense, on the other hand, uses shields and the finger’s ground to align the fields and reduce noise from the environment. Additionally, a layout for a 2-layer flexible circuit board was designed to reduce short traces.
- **Self-Contained:** eRing is connected to a laptop via a USB cable to supply the ring with power and handle the data transfer. PeriSense is self-contained. It is equipped with a battery and a Bluetooth module.
- **Evaluation:** The eRing prototype was just tested with a reasonable set of gestures. The test set was only recorded from one author of the paper. The test should only show the general feasibility of the idea. In this work, we have conducted an extensive evaluation of PeriSene. First, the technical evaluation is more accurate and reproducible. On the other hand, we evaluate different interaction techniques. For this purpose, we have recorded and analyzed examples from several users.

In another paper [56], we have already briefly introduced PeriSense. We have evaluated whether it is possible to reconstruct finger movement from the capacitive values. However, this was not related to gesture recognition. In this paper, we present the full technical specifications of the ring and evaluate for finger gesture recognition.

## 3. PeriSense

The following section describes PeriSense, in particular, its working principles and its technical properties. After describing the basic concepts of the prototype, details on its hardware design are given. The section concludes with an evaluation of its capacitive sensor properties.

### 3.1. Concept

Capacitive proximity sensing allows the detection of conductive objects. This is achieved by measuring the effect on the electrical field created by an electrode. This technology is widely applied in different application [57,58,59]. In the context of human–computer interaction, it finds application, for example, in gesture recognition (see, e.g., in [27,33,44,60,61,62]), activity recognition (see, e.g., in [15,63,64,65,66]), or touch recognition (see, e.g., in [67,68,69]).

The concept of PeriSense is based on arranging four capacitive electrodes on a finger ring and employing capacitive proximity sensing in loading mode [58] to quantify the distances to the adjacent fingers. Measuring proximity between conductive objects can be achieved effectively by capacitive sensing in several different modes [70]. We apply the loading mode, its purest form, which drives a single electrode with an oscillating signal, leading to the periodic charging and discharging of the respective electrode. Such an electrode acts as one plate of a capacitor while the surrounding conductive objects in its environment act as the opposite plate connected to ground. The capacitance itself is proportional to the size of the electrode and inversely proportional to the displacement to its complement. By approaching the electrode with such a conductive object, its charge time increases consequently as there is more energy to store at a higher capacity. The capacitance can thus accordingly be computed by measuring those changes in frequency. As in such a scenario, the human body serves as the ground electrode; the human body needs to share the same ground potential as the electronic sensing device.

**Figure 2:**
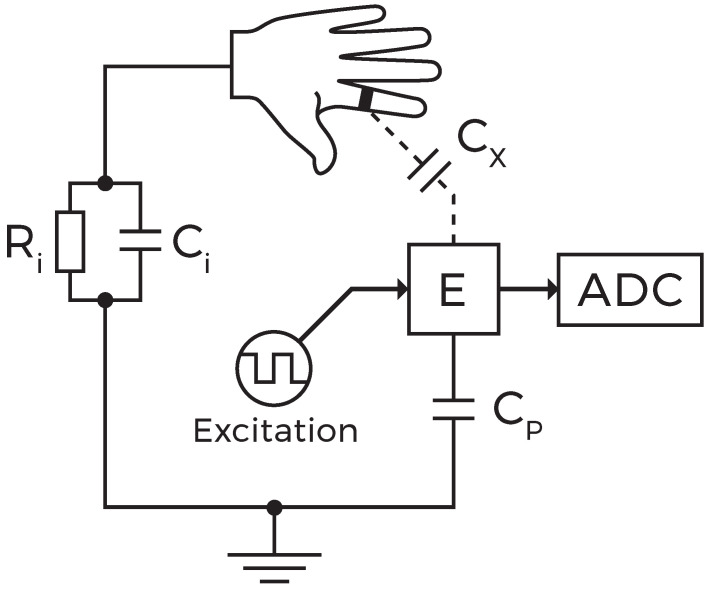
Lumped circuit model of measuring capacitance $C_{x}$ at a single electrode *E*.

Figure 2 depicts a simplified lumped circuit model for a single electrode *E* and exemplifies the conception along with its inherent limitations. The inserted capacitance symbols are no dedicated components but refer to the actually measured capacitances and are simplifications for a comprehensible presentation. $C_{x}$ is the capacity that relates to the proximity between an electrode and neighboring fingers, which is in principle a capacitor with the electrode being one plate and a grounded conductive object in the surroundings its counterpart.

The surface capacitance $C_{x}$ is proportional to the area of overlap between the electrode and the opposed conductive object. The relationship between capacitance *C*, the area of overlap *A*, and the object proximity *d* is given by $C=\epsilon_{0}\frac{A}{d}$, with $\epsilon_{0}$ being the electric constant. Thus, the surface capacitance is proportional to the exposed surface area of the electrode. Given a particular measurement range of a capacitive sensor, its lower end capacitance determines the theoretical maximal measurable distance. In practice, this distance is even lower as measurement resolution and noise impose much more significant impact at small capacitances.

Furthermore, $C_{i}$ is the intrinsic human body capacitance and $C_{p}$ the inevitable parasitic capacitance present in every physical circuit. In principle, device pins and the presence of ground return lines in a circuit design add parasitic capacitances that are summed up in the measured total capacitance. Clean signal flow and short traces are therefore essential requirements for designing the electrical layout. Another potential difficulty is imposed by the nonlinear relationship between proximity and measured capacity [70]. While movements near the electrode result in drastic changes in capacitive measurements, its sensitivity decreases at increasing ranges [71].

**Figure 3:**
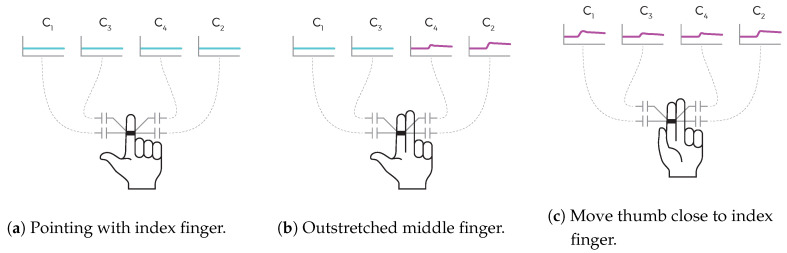
Capacitive proximity sensing working principles.

The four electrodes are arranged in a linear array on the ring to enable a form of spatial perception. Two of the four electrodes are directed sidewards. These electrodes are in practice limited to sensing only the movements of the corresponding neighboring fingers. The other two electrodes are directed towards the palm and gather proximity data below the wearing finger. Figure 3a–c illustrate this working principle symbolically. If the only outstretched finger is the index finger (Figure 3a), the capacitive sensor values are rather low. Approaching the ring with the middle finger (Figure 3b) or the thumb (Figure 3c) increases the particular capacitive sensor measurement values.

In contrast to a lot of other sensing modalities, capacitive sensing can sense a wide field of view at close distances without the need for a lens [59]. The downside is that it is not trivially possible to differentiate the causes of changes in total capacitance for a single electrode. In order to overcome this limitation, an active shield is driven at the same potential as the electrode under measurement. Although the overall sensitivity is reduced, the measurement can be blocked partially and directed in particular directions [72].

### 3.2. Hardware Prototype

As short traces and a reproducible design were crucial requirements, a layout for a 2-layer flexible circuit board was designed. This way, the copper electrodes for capacitive proximity sensing can be laid out in an editor for reliable production by a common PCB manufacturer. The copper electrodes are designed as rectangles with rounded corners, with lengths of edges ranging from 12 to 17 mm, forming areas from approximately 200 to nearly 280 mm^2^. The electrodes pointing downwards are smaller than the electrodes pointing side-wards. Although the measurable distance decreases with smaller electrodes, this limitation is mitigated as these two electrodes primarily detect finger bending. The two electrodes on the side, however, primarily measure the distance to the adjacent fingers. This distance can measure a few centimeters and is the reason why these electrodes are laid out larger.

The thickness of a copper layer is specified by 35 μm. The active shield is laid out below the electrode layer to direct the capacitive measurements outwards. The flexible base material allows for a convenient way of placing all electrical components in a 3D-printed finger ring casing. The dimensions of the ring case are a width of 22 mm, a height of 44 mm, a variable inner diameter between 18 and 22 mm (depending on the finger size and prototype), and outer diameter of 27 mm. The cases of our prototypes have a thickness of 2.5 mm in this area, with a shell thickness of 0.5 mm.

**Figure 4:**
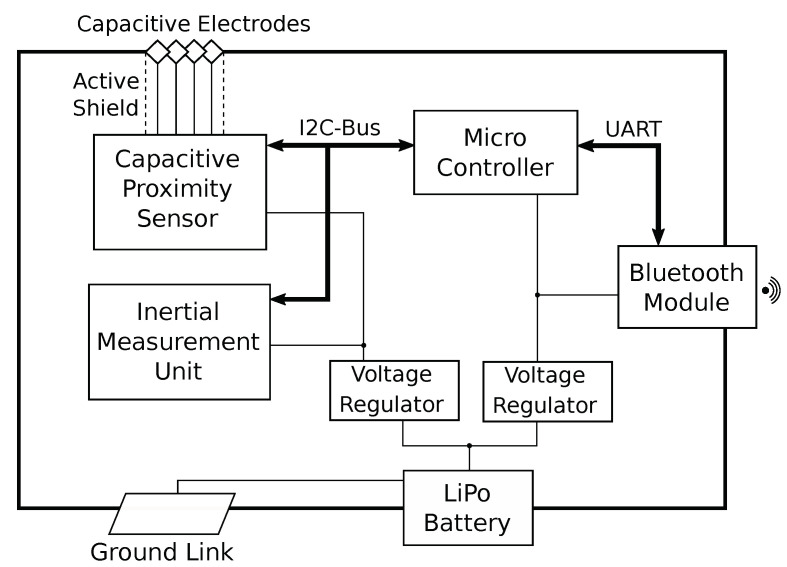
System architecture block diagram.

In order to reduce cross-talk between sensor measurements and their processing and transmitting, the flex-board is segmented into two parts with dedicated voltage regulators. The sensor segment features a 4-channel capacitance-to-digital converter for capacitive proximity sensing [73]. Each channel is connected to one of the four electrodes which are sampled in sequential order. As the ring device and the human body have to share the same ground potential for this sensing mode to function correctly, the inside of the ring is coated with a copper foil to provide an electrical connection to the worn finger. An additional inertial measurement unit (IMU) with 9 degrees of freedom [74] is equipped to augment sensor readings for gesture recognition with hand position and orientation tracking. Figure 4 provides a block diagram for illustration of the underlying system architecture.

**Figure 5:**
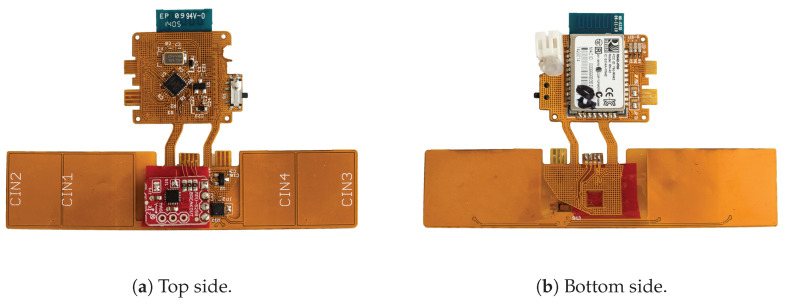
Assembled flexible circuit board.

Figure 5 shows an assembled circuit board. The top segment includes an ATmega328P for reading out the respective sensor measurements sequentially using an I2C bus and transmitting those readings with the help of a Bluetooth 2.1 module connected over UART at a baud rate of 115,200 bps. The device is driven by a small-sized LiPo battery with a capacity of 110 mAh. Consuming ~128 mA at 3.0 V on average during measurement and Bluetooth communication, a battery charge lasts for around an hour of continuous usage. By disabling Bluetooth communication and using UART exclusively, average power consumption went down to 16 mA.

### 3.3. Spatial Resolution of PeriSense

In order to determine the effective interaction space of PeriSense, the spatial resolution for each electrode was computed by measuring the capacitive proximity in a grid surrounding the ring. The spatial resolution is defined in [75] as “the smallest distance between two identical objects that produce a signal with a measurable difference compared to the signal they would produce if they were superimposed”. The computation of the spatial resolution was based on the procedure for flat electrodes employed in [71]. Therein, the spatial resolution is determined by computing the mean μ(d) and standard deviance σ(d) across measurements of the same true proximity *d* and measuring the maximum pairwise distance between the border measurements that falls into the range defined by (μ(d)−σ(d),μ(d)+σ(d)).

Opposed to the usage of flat electrodes, in this case, the electrodes are bent on a ring. This is the reason why measurements were gathered in a grid and not just at one-dimensional distances. For each of these 2D-measurement positions *p*, a set $P_{p}$ of all samples at this position was constituted, and the mean $\mu (P_{p})$ and the standard deviance $\sigma (P_{p})$ were calculated. Subsequently, a set of all measurement positions $P_{p,s}$ was created which fell into the measurement range defined by $\left(\mu \left(P_{p}\right)-\sigma \left(P_{p}\right),\mu \left(P_{p}\right)+\sigma \left(P_{p}\right)\right)$. In this resulting set $P_{p,s}$, the maximum pairwise difference of distances to the ring-border was taken. This resulted in a one-dimensional distance projection, and therefore is analogous to the method in [71].

**Figure 6:**
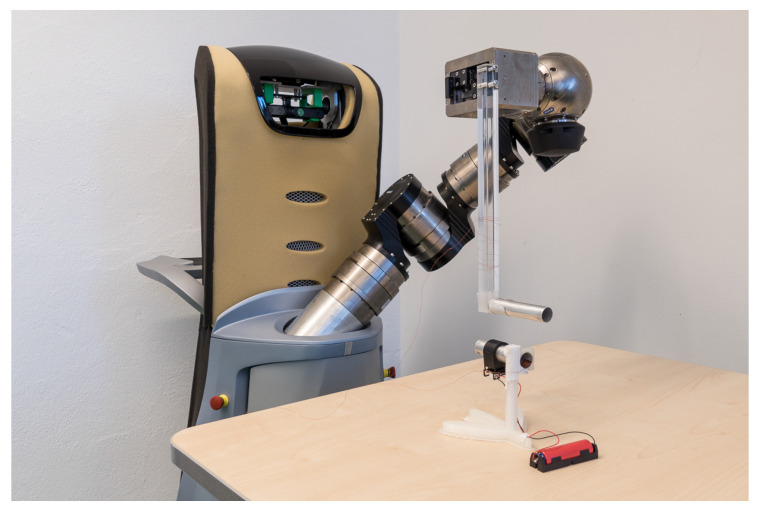
Experimental set-up for evaluation of spatial sensor resolution.

For executing such an experiment, the limb of a Care-O-Bot 3 robot was equipped with an acrylic bar of 30 cm to keep the robot’s capacitive influence as small as possible (Figure 6). At the end of the bar, an aluminum tube with a length of 10 cm and a diameter of 2 cm was mounted, simulating a neighboring human finger, similarly to the simulated arm in [71]. As the simulated finger and PeriSense have to share the same ground potential for optimal performance, the aluminum tube was connected to the exposed ground link via a small diameter wire to achieve comparable results. The ring itself was put on a 3D printed mount.

The robot traversed the measurement grid from left to right, top to bottom in steps of 5 mm. All positions refer to the center of the ring and represent the center of the aluminum probe. As the probe has a diameter of 2 cm, this accounts for the minimum measurement gap of 1 cm around the ring in the resolution plots. At each measurement position, 5s of capacitive values were read after a short settling time of 2s. This procedure lasted about 5 hours, and thus exceeded the small battery capacity of the prototype. As before, the small battery was replaced with a bigger 18650 type battery with 3.500 mAh, which was put aside on the table.

**Figure 7:**
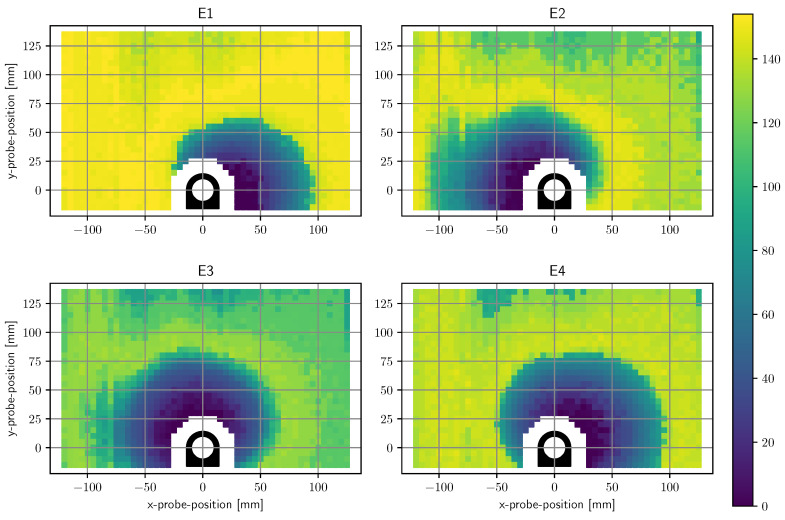
Spatial resolution for each electrode. The color represents the particular resolution in millimeters.

**Figure 8:**
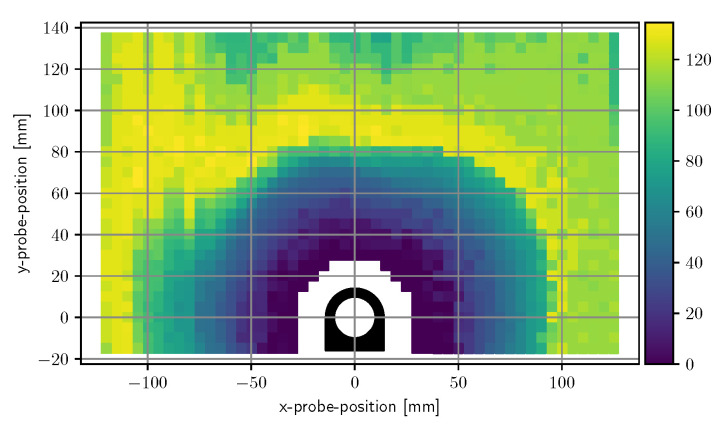
Superimposed plot of spatial resolutions from all of the four electrodes.

**Figure 9:**
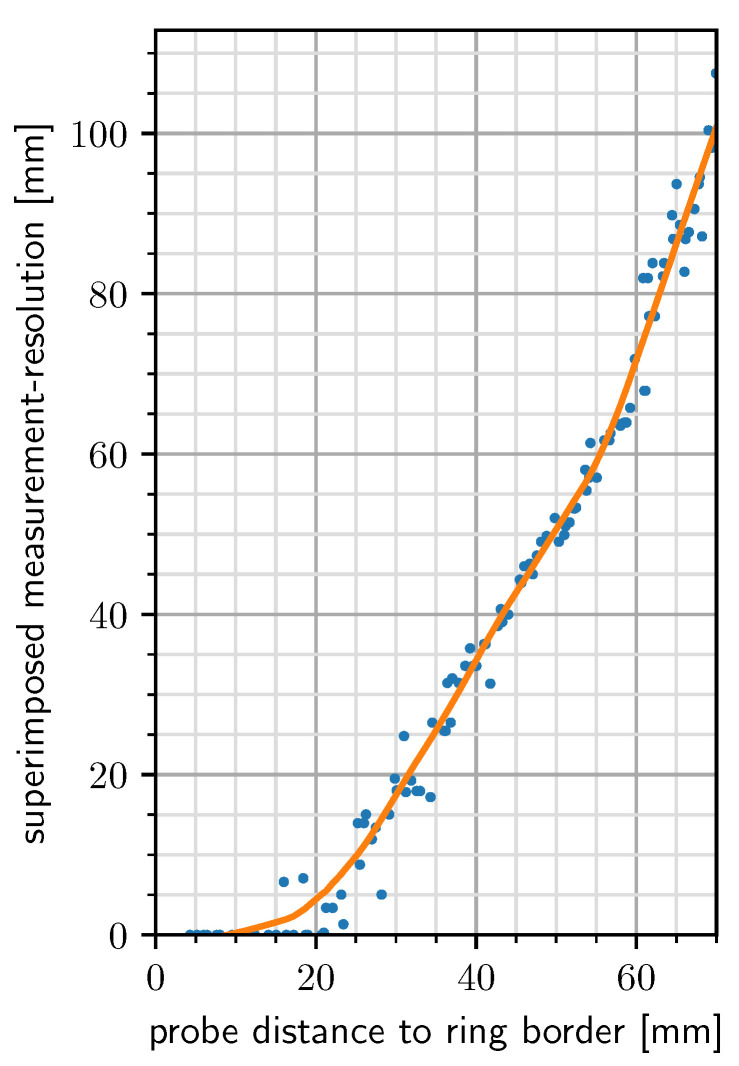
Spatial resolution at distances measured between ring-border and tube-border.

Figure 7 contains a plot of the computed resolution values of each electrode for each measurement position. At low proximities, the electrodes have a resolution in millimeter range. The figure also depicts which areas are exclusively measured by a single electrode and which areas are overlapping. Up to a displacement of approximately 5 cm most of the surrounding positions are sensed by at least two electrodes. Figure 8 shows a superimposed plot of all four electrodes by taking the minimum of each resolution. Figure 9 depicts the superimposed resolution in relation to the actual distance between ring border and probe border. It suggests that up to 1.5 cm the superimposed resolution is in the order of millimeters as the computed resolution is zero, which means no matchable measurement positions could be found in a 5 mm grid. As the relevant proximity for gestures is way above this value, no further efforts were made to reduce grid size. The curve then forms a knee upwards at around 2 cm, seems to rise linearly up to 6 cm, and increases much more afterward. While small probe distances can be resolved with a very high resolution, a probe distance of, for instance, 6 cm cannot be reliably distinguished from a distance of 12 cm.

This corresponds to the previous statement, that capacitive proximity sensing is highly nonlinear and most sensitive in close proximity. Although the range could be increased by increasing the electrode size, this would lead to a bigger form factor which is undesired. Displacements between fingers rarely exceed such ranges anyway. The plots show also that the smaller electrodes pointing downwards have a very similar resolution like the larger electrodes.

## 4. Evaluation of The Interaction Space Based on Interaction Techniques

In the previous section, we studied technical properties and determined the spatial resolution with a simulated finger. However, in reality, the measurements are influenced by use of a greater number of fingers and the hand itself. To determine the interaction space with the influence of the whole hand, we ran an evaluation based on two different interaction techniques: finger gestures and unistroke gestures drawn above the ring. First, we describe the evaluation procedure and afterward, we describe and discuss the interaction techniques and evaluation results in detail.

### 4.1. Method

In the following, we describe the general procedure of the experiments, namely, participant selection, measurement, and experiment set-up common to all experiments. More specifics of the respective evaluation method are given in the subsection of the respective interaction method.

#### 4.1.1. Participants

All experiments were performed with participants that had no previous contact with PeriSense or similar interaction devices and who could move their fingers painlessly and in an unrestrained manner. All participants received a voucher for an online store of 30 €. The group involved two invited colleagues and eight external participants acquired via flyers placed at the university campus and supermarkets close to the campus. Overall, the group consisted of 10 participants with different hand sizes (hand length between 16 and 19.8 cm (μ = 18.26 cm, σ = 1.38 cm), span width of the hand between 18.3 and 23.1 cm (μ = 20.43 cm, σ = 1.56 cm), index finger length between 6.2 and 7.5 cm (μ = 7.07 cm, σ = 0.52 cm) and gender (female = 5, male = 5, age range between 25 and 46).

The thumb and index finger circumference of each participant was between 56 and 70 mm. To enable evaluation with different finger sizes, we produced three rings with a different inner diameters (18, 20, and 22 mm). The outer sizes were kept unchanged and are also identical in regards to technical properties such as signal noise and resolution.

Two participants were left-handed and also used their right hand to gesture during the experiments. However, none of them report any difficulties in performing the gestures with the right hand after practicing the gestures.

#### 4.1.2. Measurement Logging

During each experiment, 14 sensor values (four capacitive electrodes and 9 dimensions and a temperature reading from the IMU) were logged into a CSV file along with associated timestamps (time in milliseconds starting from startup of the ring), sample ids (an increasing counter), requested gesture labels (if the program requested the user to perform a specific gesture), and gesture activations (signs when the user performed the requested gesture). All measurements were captured at a rate of 100 Hz. Although we logged all those sensor values, we constrained the data to capacitive measurements, acceleration, angular velocity, and class labels for our evaluation.

#### 4.1.3. Recording Procedure

As the index finger is a primary interaction finger, PeriSense was worn on the base segment of the index finger of the right hand. Before these tests, the gestures were explained, and the participants had time to exercise them. The gestures were requested and performed in a random order. The participants were asked to move the hand and fingers into the start position of the requested gesture, to hold a second, to perform the gesture, and to again hold for a second in the end position of the performed gesture. The short breaks at the beginning and end of each gesture were necessary for the experimenter, who noted the start and end of the gesture by simultaneously pressing a key on a keyboard. During the whole experiment, the participants received no feedback of the gesture recognition result. The participants were allowed to take as much time as they needed to take a break, touch, and adjust the ring, and also relax and move the fingers freely.

The interaction techniques were performed in a single session per participant. Each session consisted of two cycles, in which the experiments were performed in the following order; finger gesture interaction followed by unistroke gestures. For each experiment, the gesture sets consists of eight gestures, and 15 samples of each gesture were recorded resulting in 240 per user and gesture set (15 samples × 8 gestures × 2 cycles). The user was able to take a break after each experiment.

#### 4.1.4. Evaluation Procedure

We performed a leave-one-out cross-validation on each of the 20 cycle sets per interaction technique where each gesture sample is tested against the remaining gestures in the set.

All tests were executed offline with segmented gestures regarding their labels in the cycle sets. The finger gestures were min–max normalized, and the unistroke gestures were standardized. We give further details in the corresponding sections. For the cross-validation, we used one nearest-neighbor (1NN) [76] classifier to decide to which class the gesture segment belongs. The applied similarity measure is a multidimensional dynamic time warping (DTW) algorithm [76] and can adapt to different speeds of performing a gesture. 1NN with DTW distance produces comprehensible and reproducible results, while still being a very robust classifier for sequences [77].

### 4.2. Finger Gestures

**Figure 10:**
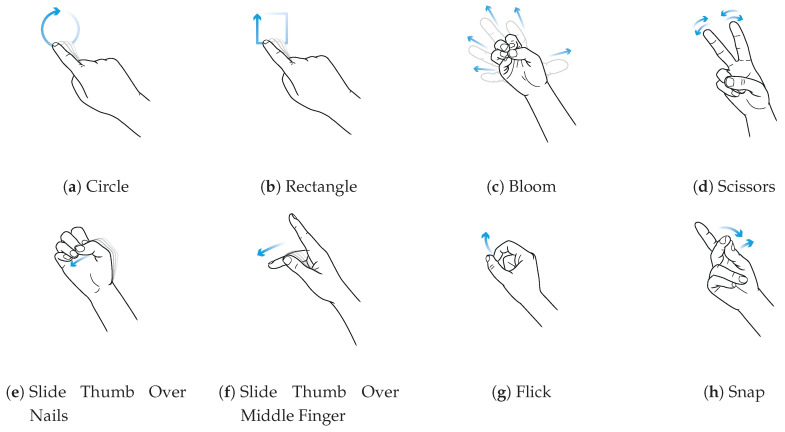
Finger gesture set used for evaluation.

**Figure 11:**
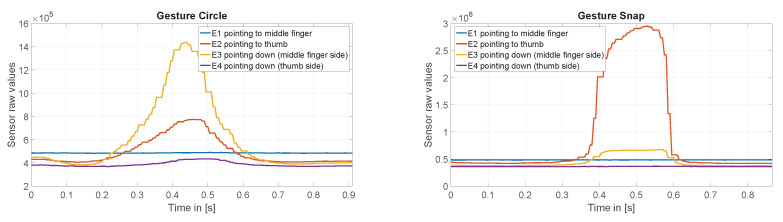
Raw values of the capacitive sensor for gestures Circle and Snap.

The finger gesture set consists of specific finger movements, depicted in Figure 10. These finger gestures allow for fine and subtle micro-interactions. To study the interaction space, we selected different finger gestures from various published gesture sets different in execution but partially similar in the resulting sensor pattern. Consequently, it is not our intention to show that we are exceptionally good at recognizing a particular set of gestures. Rather, we want to show where the boundaries are in the differentiation and at the same time which diverse gestures can be distinguish. Therefore, we have chosen a mix of gestures that, on the one hand, generate similar patterns, and on the other hand, involve different finger movements. This should give an impression of which interactions are possible and which are not. In this manner, gestures Bloom and Flick the same index finger movement. Gestures Circle and Rectangle consist of a very similar pattern. It is also notable that the index finger only draws these two gestures without moving the hand. Figure 11 shows exemplary the raw values of the capacitive sensor for gesture Circle and Snap.

For the gesture recognition, we applied a min–max normalization and conducted two cross-validations. First, we used only the four capacitive measurements, and in the second run we additionally used the acceleration and gyroscope measurements.

#### 4.2.1. Results

**Figure 12:**
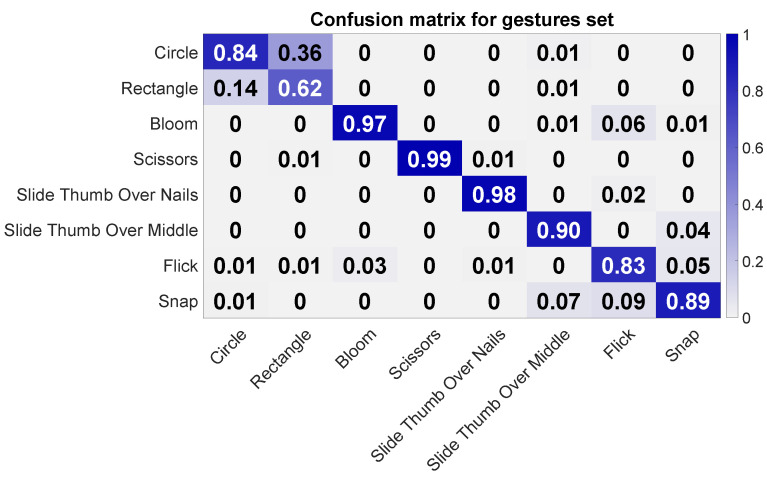
Confusion matrix for the finger gesture set using only capacitive measurements. The columns refer to the actual class, and the rows to the assigned ones during the classification.

The leave-one-out cross-validation test using only capacitive measurements revealed an average accuracy of 0.88 (σ = 0.017). Two users approached only 0.85 and 0.86, while one user rendered an accuracy of 0.91. As can be seen in Figure 12, most confusion occurred between gestures Rectangle and Circle, where Rectangle was mostly confused with Circle. Gestures Bloom, Scissors, and Slide Thumb Over Nails achieved an accuracy of 0.97 and higher. Gesture Snap had some confusion with Flick and Slide Thumb Over Middle Finger. Additionally, gesture Flick also shows some confusion with Bloom. Interestingly, Flick was two times more confused with Bloom than Bloom with Flick.

**Figure 13:**
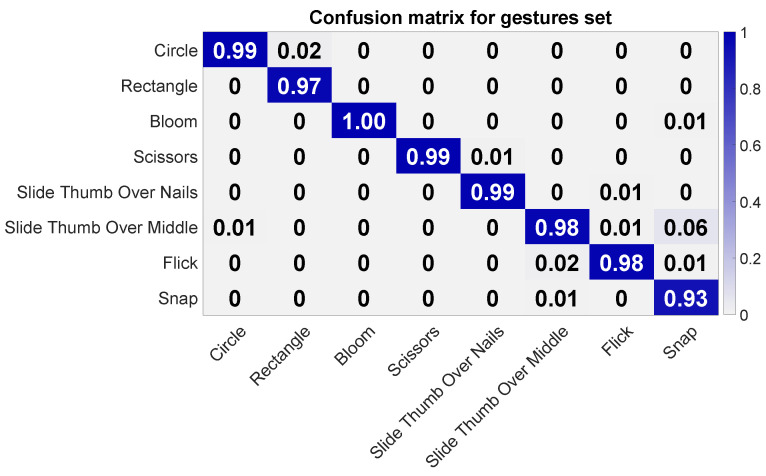
Confusion matrix for the finger gesture set using capacitive measurements, acceleration, and gyroscope data. The columns refer to the actual class, and the rows to the assigned ones during the classification.

In order to study if the usage of the motion sensor data can reduce these confusions, we ran a further cross-validation test and augmented the capacitive measurements with the three-dimensional acceleration and gyroscope values. This test resulted in an average accuracy of 0.98 (σ = 0.007). As Figure 13 shows, all confusions are resolved except for gesture Snap, which shows some confusions with Slide Thumb Over Middle Finger.

#### 4.2.2. Discussion

Gestures Bloom, Scissors, and Slide Thumb Over Nails provide a robust pattern. Moreover, gestures Slide Thumb Over Middle Finger and Snap show reasonable results. The patterns of Circle and Rectangle are very similar and primarily indicated by a short contact of electrodes 2 and 4 with the middle finger. Applying information from the motion sensor solves this issue because it adds information about directional changes in finger movement. For gesture Rectangle, the finger movement stops at each corner, and for Circle, it is a smooth ongoing movement. Despite the high confusion between Circle and Rectangle using only capacitive measurements, there is barely any confusion with other gestures. Confusion between gestures Flick and Snap comes from variations in the execution of Snap. Sometimes the participants started Snap with index and middle finger straight, bending the middle and moving backward the index finger. This results in almost the same pattern in the capacitive measurements. Here, again, using the additional acceleration and gyroscope data can solve this confusion issue. Only the confusion between Snap and Slide Thumb Over Middle remains. In general, regarding the interaction space we can conclude that PeriSense produces proper distinguishable patterns for finger gestures based on capacitive measurements. However, similar motion and variations in execution can lead to confusions. Furthermore, we assume that smaller and more electrodes could also reduce confusions. The quite large electrodes seem to hinder the sensing of smaller finger displacements along the electrodes. By employing data from the accelerometer and gyroscope for classification, these confusions can be diminished. This allows a broad definition of finger gestures used with PeriSense enabling a variety of applications.

### 4.3. Unistroke Gestures

**Figure 14:**
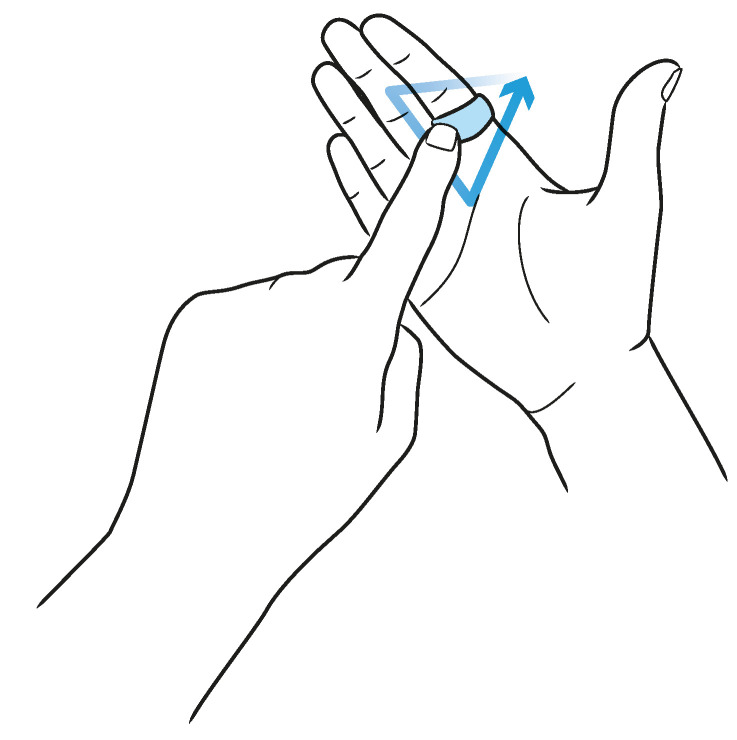
Drawing unistroke gestures above PeriSense.

**Figure 15:**
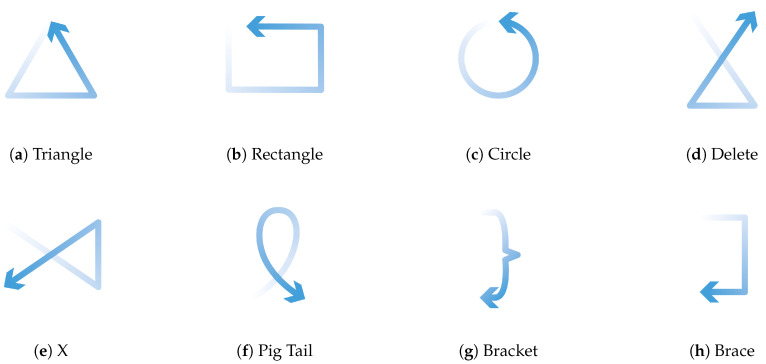
Unistroke gestures used for evaluation. The gestures start at the thin slightly transparent end and follow the direction of the arrow and end at the arrow.

In Section 3.3, we showed that PeriSense is able to sense the change of conductive objects reliable up to 2.5 cm with a resolution between 5 and 7 mm. That is why we want to evaluate if this capability can be used for the device regarding interactions extending the interaction space by another interaction technique. For this purpose, we want to test if we can detect small unistroke gestures drawn with the other hand’s index finger above the ring. Figure 14 depicts an example for such an interaction technique. In order to study how well we can distinguish similar gestures, we selected eight unistroke gestures from the work in [78]. These are depicted in Figure 15. The unistroke gestures Circle, Rectangle, and Triangle are executed counterclockwise; Pig Tail is similar to Circle in different orientation; X is similar to Triangle in different orientation; and Delete is a rotated and mirrored version of X. Gestures Bracket and Brace differ from the remaining, but these are both quite similar when drawing on a small space with a finger.

These gestures are not directly drawn on the ring surface but at a proximity of approximately 5 to 30 mm above the ring. The right hand is to be held as shown in Figure 14, and the gestures are drawn with the left hand’s index finger where the downward direction of the gesture is towards the palm. The active writing area is directly above the ring. Although most users were right-handed, they reported no difficulties in drawing with their left hand.

As when wearing PeriSense with the right hand it is not moving during the unistroke execution, we use only the capacitive measurements without motion data for the gesture recognition. Additionally, we omitted the right electrode because it is covered by the middle finger. Consequently, for this experiment we use only the electrode measurements of the remaining three electrodes for the unistroke gesture recognition. Due to variances in proximity between PeriSense and the drawing finger, we standardized each sample by centering to the mean and scaling to unit variance in order to stress the pattern and normalize its amplitude.

**Figure 16:**
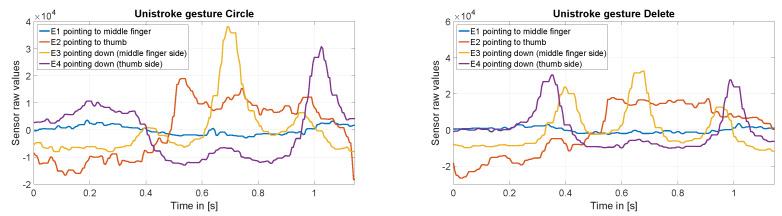
Raw values of the capacitive sensor for unistroke gestures Circle and Delete.

Figure 16 shows exemplary the raw values of the capacitive sensor for unistroke gesture Circle and Delete. As the offsets of the sensor values vary much more than the amplitudes, we have subtracted the mean value for each sensor value to make the amplitude visible and comparable.

#### 4.3.1. Results

**Figure 17:**
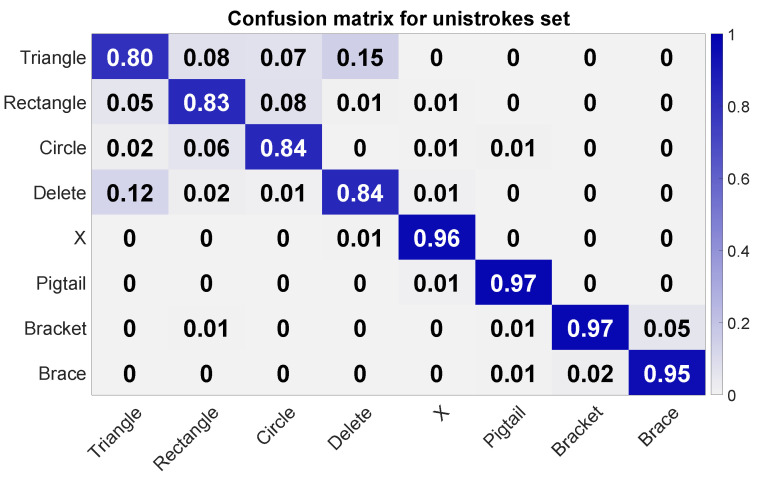
Confusion matrix for the unistroke gesture set. The columns refer to the actual class, and the rows to the assigned ones during the classification.

The leave-one-out cross-validation over each single circle data set revealed an average accuracy of 0.9 (σ = 0.031, minimum accuracy of 0.83, and maximum accuracy of 0.93). The confusion matrix in Figure 17 shows a reasonable accuracy of 0.95 and more for gestures Bracket, Brace, Pig Tail, and X. Despite the good precision of Bracket and Brace, there is some confusion between these two unistrokes. A big confusion cluster can be identified for Triangle, Rectangle, Circle, and Delete. Most confusion is between Triangle and Delete. Triangle was also often confused with Rectangle. Circle was primarily confused with Rectangle and Triangle. The same can be observed for Rectangle.

#### 4.3.2. Discussion

The test revealed that PeriSense is also able to detect nearby gestures performed with the other hand, in this concrete case two-handed unistroke gesture input. In general, this input technique generates well-distinguishable patterns for most gestures. The big confusion group of Triangle, Rectangle, Circle, and Delete seems to arise from the one-dimensional electrode arrangement, in particular for the confusion between Triangle and Delete, as it is not possible to detect the direction of movement along the electrode (Figure 16). The ability of detecting multiple finger gestures and device around interactions increases the interaction space and, thus, the variations of applications.

## 5. Example Applications

So far we studied the gesture interaction space of PeriSense. Following a short proposal of an example recognition pipeline, we showcase two use cases in which PeriSense could be used. These use cases also employ the inertial measurement unit, which enables tap recognition and the motion tracking of the whole hand in addition to the gesture recognition.

### 5.1. Gesture Recognition Pipeline

**Figure 18:**
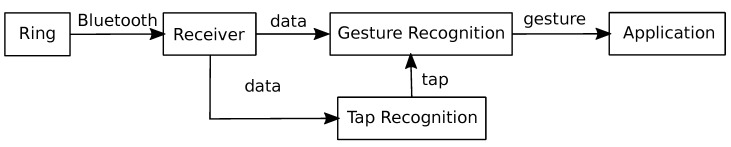
Gesture recognition pipeline for an abstract application.

Given that the ring device only forwards its sensor readings via Bluetooth, a receiving counterpart is necessary for further processing. This can be either implemented on a computer or a smartphone utilizing its native Bluetooth integration. The gesture recognition module then accepts the forwarded sensor readings as an input and will output gesture notifications to an application after successfully classifying a known gesture. An additional tap recognition can then enhance the gesture recognition module by processing inertial measurement data. This way, a gesture can be announced by double-tapping the ring with the thumb, which then activates the gesture recognition module. Figure 18 depicts a simple flow chart of this system for a better illustration.

### 5.2. Smart Home Interaction

Smart homes contain various networked devices which can be integrated with each other and combined to complex home automation scenarios. The user experience can be improved by flexible interaction systems that enable the user to interact with any device from anywhere at home in their daily life. The ability to be worn throughout the day makes PeriSense a suitable interaction device in this context. Frequent automation scenarios, like switching on a light or shutting down all devices when leaving home, could be tied to representative gestures to make them available to the user anywhere throughout their daily routine. Furthermore, the ability to draw unistroke gestures above the ring could be used as textual input, e.g., to enter a pin for door opening.

In our example, showroom application the compass of the motion sensor is used to determine the direction in which the user is pointing with the ring, while each device needs to be linked to a distinct compass direction. By pointing in the specified direction and double-tapping the ring with the thumb, the corresponding device is selected, a gesture is executed, and the corresponding action is triggered by the system. This can have a variety of outcomes: roller blinds can be raised or lowered by pointing to the windows and a floor lamp can be turned on/off by pointing in the corner or a ceiling light by pointing upwards. More complex interactions were implemented, for instance, for a smart mirror to enable manipulation of display elements and radio for controlling stations and volume.

To minimize errors, the occurrence of very similar gestures have to be avoided by selecting distinguishable gestures for this interaction mode. By including the application’s context in gesture handling, nonsensical gesture input can be either dropped or reinterpreted. For instance, performing the “volume-up” gesture while the radio is still turned off, can additionally turn on the radio beforehand.

### 5.3. Head Mounted Displays

The current trend towards augmented and virtual reality also raises the demand for new input modalities. Existing technologies make use of camera technology or dedicated input controllers for detection of hand input and gestures. PeriSense could achieve similar results but does not suffer from occlusion or light conditions and leaves the user with free hands. The ring could serve to detect directional changes and movements via established motion-sensing techniques. Capacitive sensing can complement this by being able to sense a variety of interactions. For example, the ring could be used to detect interaction inputs, by detecting gestures, different interaction modes, by touching and holding different electrodes directly, continuous inputs, by using the distance between two fingers as pinch-style interaction, as well as text input, by making use of unistroke interactions. This could serve as a basis for complex interaction patterns for augmented and virtual reality applications as well as games.

## 6. Conclusions and Future Work

We presented PeriSense, a multi-finger gesture interaction device in the form of a ring utilizing capacitive sensing. We determined the effective resolution at different distances and based on two different interaction techniques, we studied how well different gestures can be detected and distinguished in order to determine the interaction space.

Initially, we assumed large electrodes with higher sensing resolution at larger distances would result in an advantage regarding recognition accuracy. However, we estimate that decreasing the electrode size while increasing the number of electrodes can be an advantage as most of the gestures account for changes in close proximity. Currently, the electrodes are arranged in one line across the ring’s outer surface. Arranging them in two dimensions on this surface could lead to noticeable increases in sensor performance, in particular, for unistroke input. Smaller electrodes additionally enable smaller ring forms. This would foster wearing comfort and flexibility of the wearing finger as the current prototype limits the finger bend flexibility depending on the finger length. A trade-off between electrode size and its sensor range has to be taken though.

A technical limitation is currently the power consumption of PeriSense. This is mainly founded in the used Bluetooth module consuming around 85% of the total power usage. The power consumption of our prototype without the Bluetooth module is ~50 mW and with the Bluetooth module enabled at about ~380 mW. Using a Bluetooth low-energy module (BLE), the energy consumption could be reduced by a factor of 10. However, BLE is not designed for continuous data transmission at a relatively high frequency. We have to study whether a sufficiently rate (minimum 60 Hz) can be reached and how much power can be saved. Additionally, utilizing a single controller for I2C and RF communication will lead to a much more energy friendly design. Additional power saving routines could be implemented. For example, the low power wake on motion interrupt of the MPU9250 motion sensor could be used to suspend and wake up the microcontroller and its attached sensors. Furthermore, it is also inefficient to send all data points to an external device continuously. A low-cost prefiltering algorithm could help to avoid sending noise and meaningless data, and would thereby reduce the number of transferred packages and the overall power consumption of the communication module.

After showing the promising potential of PeriSense to enable diverse and versatile multi-finger gesture interactions with a ring, we want to implement an online gesture recognition pipeline. Furthermore, we want to test and evaluate PeriSense in the context of real-life applications such as smart home control or as input controller for head-mounted displays. In this context, we also want to test if using additional features or other classifiers for gesture recognition can improve the general accuracy.
